# Highly Sensitive Immunochromatographic Identification of Tetracycline Antibiotics in Milk

**DOI:** 10.1155/2015/347621

**Published:** 2015-11-25

**Authors:** N. A. Taranova, A. S. Kruhlik, E. A. Zvereva, V. V. Shmanai, I. I. Vashkevich, D. A. Semyonov, S. A. Eremin, A. V. Zherdev, B. B. Dzantiev

**Affiliations:** ^1^A.N. Bach Institute of Biochemistry, Research Centre of Biotechnology of the Russian Academy of Sciences, Leninsky Prospect 33, Moscow 119071, Russia; ^2^Institute of Physical Organic Chemistry, Surganov Street 13, 220072 Minsk, Belarus; ^3^Institute of Bioorganic Chemistry, Acad. Kuprevich Street 5/2, 220141 Minsk, Belarus; ^4^Chemical Department, M.V. Lomonosov Moscow State University, Leninskie Gory, Moscow 119991, Russia

## Abstract

A rapid immunochromatographic assay was developed for the control of tetracycline (TC). The assay is based on the competition between immobilized TC-protein conjugate and TC in a tested sample for binding with polyclonal anti-TC antibodies conjugated to colloidal gold during the flow of the sample along a membrane strip with immobilized reactants. Conjugation of colloidal gold and the total immunoglobulin (IgG) fraction of polyclonal antibodies was used to increase the assay sensitivity to ensure low content of specific antibodies in the conjugate. This allowed effective inhibition of free TC and conjugate binding in the strip test zone. Photometric marker registration allows control of the reduction of binding, thereby enhancing detection sensitivity. The proposed assay allows TC to be detected at concentrations up to 20 ng/mL, exceeding the limit of detection of the known analogues, in a wide working range (more than two orders) of 60 pg/mL to 10 ng/mL, ensured through the use of polyclonal antibodies. The assay time is 10 min. The efficiency of the designed assay is shown to identify TC in milk; the degree of recovery of TC ranges from 90 to 112%. The precision of the concentrations measurements was no more than 10%.

## 1. Introduction

**Figure 1:**
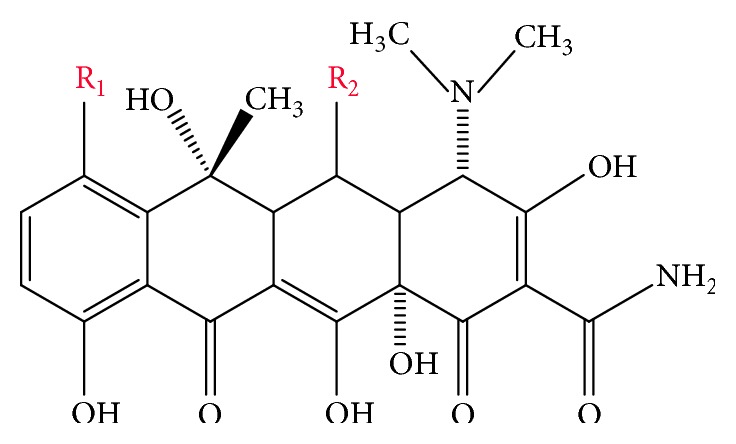
The overall structure of tetracycline antibiotics: tetracycline (R_1_, H; R_2_, H); chlortetracycline (R_1_, Cl; R_2_, H); oxytetracycline (R_1_, H; R_2_, OH).

Tetracyclines (TCs) are a group of broad-spectrum antibiotics representing polyketones in their chemical structure [1]. By blocking the binding of transferred ribonucleic acid aminoacyl to ribosome, they inhibit protein synthesis in bacterial cells [2, 3]. TCs are widely used in veterinary medicine, both for therapeutic and preventive purposes, due to their high activity towards a large number of Gram-positive and Gram-negative bacteria, small doses, and broad spectrum of action. In addition, TCs are used as growth promoters of animal body weight, destroyers of pathogens in drinking water sources, fodder, and food, and phytopathogenic agents in crop production [4]. The most widely used tetracycline antibiotics are tetracycline (TC), chlortetracycline, and oxytetracycline (Figure 1).

Because of their intensive and diverse uses, tetracyclines may enter the human body not only in the treatment of diseases but also with food. This can cause toxic and allergic effects, dysfunction of the gastrointestinal tract, renal insufficiency, and deformation of mucous tissues [5, 6]. The emergence of bacteria resistant to tetracycline antibiotics is due to their massive clinical, veterinary, and agricultural use [7]. It is therefore important to monitor the presence of tetracyclines and other antibiotics in food.

Most countries of the world have regulations concerning acceptable levels of tetracyclines in food. According to the regulations of the Customs Union, the concentration of tetracyclines in milk, dairy products, meat, and prefabricated meat products should not exceed 10 ng/mL (ng/g) in Russia [8]. The European Commission established maximum residue levels (MRLs) for the amounts of tetracyclines, namely, oxytetracycline, chlortetracycline, and their stereoisomers, of 100 pg/g for meat, 600 ng/g for kidneys, 200 ng/g for eggs, and 100 ng/g for milk [9]. Tetracycline is not allowed in some food products (honey and baby food); that is, the level of tetracycline should be below the detection threshold of the recommended analytical methods. In the United States, the maximum permissible level of tetracycline is 300 *μ*g/g for meat, 6 *μ*g/g for liver, and 12 *μ*g/g for kidneys [10]. In China, MRLs for the total content of tetracycline, hydrochloride, and oxytetracycline are 50 ng/mL for milk, 600 ng/g for kidneys, 300 ng/g for liver, and 100 ng/g for muscular tissue [11].

Low MRLs necessitate highly sensitive methods for detecting antibiotics. Control of TCs in food products requires rapid analysis methods and needs to be implemented directly at sampling points. However, stationary methods are still predominant in the state-of-the-art analytical practice.

Relatively simple and low-budget microbiological methods are widely used to identify TC residues in foods [12, 13]. However, they lack specificity (different classes of antibiotics are identified) and are time-consuming (1.5–3 h). Methods based on liquid chromatography are also used to identify TCs [14, 15]. These methods are highly specific and highly sensitive, but they are also time-consuming; moreover, they require large amounts of solvent and an expensive, specialized equipment base. None of these methods allow field analysis to be performed.

Methods based on the use of specific cell receptors [16, 17], including their rapid variants, have gained widespread currency because of the difficulty of obtaining antibodies specific to TCs. TC receptor tests are characterized by their rapidity (10–20 min), but the test system needs to be incubated in a constant-temperature bath at 45°C.

A number of immunochemical detection methods for TCs have also been successfully developed. Implementation of these methods does not require expensive equipment and is therefore of interest for mass contamination screening. The most common methods of microplate enzyme immunoassay (ELISA) for TCs [18–20] are implemented in a laboratory environment, but with the use of relatively inexpensive and easy to use instrumentation. Unlike ELISA, immunochromatography (IC) test systems are nonlaboratory analytical tools. All processes in the IC test system are carried out without operator intervention or additional devices: contact of the test strip with the sample initiates all subsequent interactions. There are several options for IC test systems to identify tetracycline antibiotics based on the use of monoclonal antibodies. Existing test systems allow visual detection of TCs at 30 ng/mL and above [21, 22], but this satisfies only part of the above regulations. Therefore, the creation of a highly sensitive IC assay is an extremely necessary and urgent task.

Two approaches for lowering the limit of detection of LFTS are implemented in this paper. The first approach is based on the coating gold nanoparticles (GNPs) by polyclonal antibodies allowing low content of specific antibodies against TC in the conjugate [23]. As we have previously shown [24], for such conjugates, less antigen in the sample causes saturation of the antibody valency and binding inhibition in the strip test zone. The second approach is based on the use of video digital detectors to register quantitatively decrease of the label binding. These detectors [25, 26] allow the quantization of the reduction in the intensity of coloration of the strip test zone and identification of the presence of antigen in the sample for concentrations that do not cause the complete disappearance of coloration.

Thus, the aims of the work were to obtain and characterize GNP conjugates with polyclonal antibodies against TC, to apply the described above approaches for immunochromatographic assay of TC, to characterize sensitivity of the produced test strips, and to study their application for the identification of TC in milk.

## 2. Experimental

### 2.1. Chemicals

Sodium azide, bovine serum albumin (BSA), 3,3′,5,5′-tetramethylbenzidine (TMB), dimethyl sulfoxide (DMSO), formaldehyde, Tween-20, and Triton X-100 were obtained from Sigma (St. Louis, MO). Gold chloride and tetracycline hydrochloride (TC-HCl) were obtained from Fluka (Buchs, Switzerland). Sephadex G-25 was obtained from MP Biomedicals (Santa Ana, CA). The preparation used in this work comprised the anti-TC rabbit sera and immunoglobulin (IgG) fraction described in [23]. Goat anti-mouse IgG antibodies were purchased from Arista Biologicals (Allentown, PA). Peroxidase-labeled anti-mouse immunoglobulins were from the Gamaleya Institute of Microbiology and Epidemiology, Russia. All other chemicals (salts and solvents of analytical grade) were from Chimmed (Moscow, Russia).

All solutions for synthesis were prepared using water that was purified using Milli-Q system (Millipore, Bedford, MA, http://www.millipore.com/). Stock solutions of TC (1 *μ*g·mL^−1^) were prepared in water and stored at −20°C.

### 2.2. Tetracycline-BSA Conjugate Synthesis

The procedure in [20] was amended and applied. In this procedure, 30 mg of BSA was dissolved in 2.0 mL of H_2_O and 26 mg TC-HCl solution was added to 200 *μ*L DMSO and 120 *μ*L of 37% aqueous formaldehyde solution (BSA molar ratio : TC : formaldehyde = 1 : 120 : 3,600) and stirred at room temperature overnight. The product was purified from unreacted low-molecular compounds by means of gel permeation chromatography on the Sephadex G-25 carrier in phosphate-buffered saline (PBS, 50 mM, pH 7.4, with 0.1 M NaCl). Spectrophotometry was used to evaluate the composition of the TC-BSA conjugate. The resulting product was stored at 4°C.

### 2.3. Microplate Enzyme Immunoassay for TC

Analysis was carried out using the same procedure described in [24]. The TC-BSA conjugate was immobilized in microplate wells (4 *μ*g mL^−1^ in PBS, 100 *μ*L per well) and incubated overnight at 4°C. The stock antibiotic solution was diluted in PBS containing 0.05% Triton X-100 and 1% BSA (PBST) to obtain a series of solutions in the range of 100 to 0.14 ng mL^−1^ and added to the microplate wells (50 *μ*L per well). The antibodies were diluted in PBST (dilution 1 : 40,000 from the initial antiserum volume), and the microplate was incubated for 1 h at 37°C. After washing with PBST, peroxidase-labeled anti-mouse antibodies were added (dilution of the commercial product in PBST was 1 : 6,000, 100 *μ*L per well) and incubated for 1 h at 37°C. Finally, the microplate was washed three times with PBST and once with distilled water.

To detect the activity of the bound peroxidase label, a substrate solution containing 0.42 mM of TMB and 1.8 mM of H_2_O_2_ in 0.1 M sodium citrate buffer, pH 4.0, was added (100 *μ*L per well). After 15 min of incubation at room temperature, the reaction was stopped with 1 M H_2_SO_4_ (50 *μ*L per well). The optical density of the reaction product was measured at 450 nm using a Zenyth 3100 microplate reader (Anthos Labtec Instruments, Salzburg, Austria).

### 2.4. Preparation of Gold Nanoparticles

Gold nanoparticles (GNPs) with an average diameter of 30 nm were prepared according to the protocol described in [25]. Briefly, 1.0 mL of a 1% water solution of HAuCl_4_ was added to 97.5 mL of water. The mixture was heated to reflux, and 1.5 mL of 1% sodium citrate solution was added. After refluxing for 30 min, the preparation was cooled and then stored at 4°C.

### 2.5. Generation of a Flocculation Curve to Immobilize Antibodies for GNPs

The procedure in [27] was applied with modifications. In this procedure, 200 *μ*L of GNPs (*A*_520_ = 1) was placed in microplate slots and 20 mcL of antibody solutions in water at concentrations ranging from 200 to 0.5 *μ*g/mL was added. After incubation, 20 *μ*L of 10% NaCl solution at room temperature was added to each slot within 10 min, followed by measurement of *A*_580_ and the creation of its dependence on the final concentration of antibodies (flocculation curve).

### 2.6. Synthesis of Conjugate of Antibodies with GNPs

Synthesis was carried out using the procedure in [27, pp. 931-932] as amended in [28]. Antibodies were added to 10 mM Tris buffer, pH 8.5. The GNP solution (*A*_520_ = 1) was adjusted with 1 M of potassium carbonate to pH 8.5–9.0, followed by the introduction of antibodies in an amount selected based on the flocculation curve and under thorough mixing. The mixture was incubated at room temperature for 15 min, followed by the introduction of 10% aqueous solution of BSA (v : v = 40 : 1) and incubation for 10 min under thorough mixing. Gold particles with antibodies immobilized on their surface were pelleted by centrifugation at 13.000 g and 4°C for 15 min. The supernatant was removed, and the residue was dissolved in 10 mМ Tris buffer, pH 8.5, with 1% BSA and 1% sucrose (TBSA) and subjected to centrifugation in the same environment. The resulting deposit was dissolved in TBSA with the introduction of sodium azide to a final concentration of 0.05% and storage at 4°C.

### 2.7. Sizing of GNPs and Their Conjugates with Antibodies

To characterize the particle size, images of the GNPs were obtained with a CX-100 transmission electron microscope (Jeol, Tokyo, Japan) at an accelerating voltage of 80,000 V and a magnification of 3,300,000. The images were processed by TotalLab software (Nonlinear Dynamics, Newcastle, UK).

A Zetasizer Nano ZSP (Malvern, UK) was used to evaluate the hydrodynamic diameter of free GNPs and their conjugates. Dynamic light scattering (DLS; 10 per sample) was measured at 20°C with the preliminary incubation of samples. The angle of light scattering was 103°.

### 2.8. Determination of the Amount of Antibodies Adsorbed on the Surface of GNPs

Polarization fluoroimmunoassay was used to compare concentration of antibodies in the formulation added for immobilization and the formulation of unbounded antibodies. Formulations were characterized by binding with the BSA-TC conjugate.

The TC-BSA conjugate was immobilized in microplate wells (5 *μ*g mL^−1^ in PBS, 100 *μ*L per well) and incubated overnight at 4°C. After washing with PBST, the anti-TC antibodies were diluted in PBST to obtain a series of solutions in the range of 10 to 0 *μ*g mL^−1^. After centrifugation, antibody solutions and supernatants were added to the microplate wells (100 *μ*L per well), and the microplate was incubated for 1 h at 37°C. Subsequent reaction with peroxidase-labeled anti-rabbit antibodies and measurement of the peroxidase activity associated with the label was carried out as described above (see Section 2.3). Optical densities, measured for standard antibody solutions, and supernatants were compared.

### 2.9. Production of Immunochromatographic Tests

The Mdi Easypack (Advanced Microdevices, Ambala Cantt, India) kits of membranes were used to manufacture the immunochromatographic tests. They included a plastic support, working nitrocellulose membrane CNPC with a 12 *μ*m pore size, GFB-R4 separation membrane, РТ-R7 glass fiber membrane, and АР045 adsorption membrane.

Reagents were immobilized on membranes using an IsoFlow automated dispenser (Imagene Technology, Hanover, NH). The test zone was formed by the TC-BSA conjugate and the control zone was formed by the goat anti-mouse IgG. The following concentrations and immobilization media were used: TC-BSA conjugate, 1.0–2.0 mg·mL^−1^ in PBS; and goat anti-mouse IgGs, 1 mg·mL^−1^ in PBS. One microliter of both solutions was applied per centimeter of strip width. After the dispensing, the membrane was dried at room temperature for at least 20 h. The conjugate of GNPs with antibodies at a dilution corresponding to *D*_520_ = 4.0 was spotted onto a glass fiber membrane. The conjugate load was 32 *μ*L for 1 cm of strip width. The glass fiber membrane was then dried at room temperature for at least 20 h.

After the assembly of membrane components, the obtained sheets were cut with Index Cutter-1 (A-Point Technologies, Allentown, PA) into test strips of 3.5 mm in width. The test strips were hermetically packed in laminated aluminum foil bags containing silica gel as the desiccant using a FR-900 miniconveyor. The cutting and packing were carried out at room temperature in a special room with a relative humidity under 30%. The packed test strips were stored at room temperature.

### 2.10. Immunochromatographic Assay Procedure

Milk samples were purchased from the local market. Spiked milk samples were prepared by mixing stock TC solutions and pretested TC-free milk samples.

The assay was performed at room temperature. Pure and spiked milk samples were diluted with PBS, *V*_milk_ : *V*_PBS_ = 4 : 1. A test strip was vertically submerged into an analyte solution or a milk sample for 10 min, corresponding to the time required for the fluid front to migrate along the entire length of the working membrane.

### 2.11. Registration and Processing of Immunochromatographic Assay Data

The binding of the label in the test and control zones was recorded with the use of a CanoScan LiDE 90 scanner (Canon, Tokyo, Japan) followed by digital processing of the images with TotalLab software (Nonlinear Dynamics, Newcastle, UK). This program was used to determine the spot boundaries, sum up the intensities of all pixels belonging to a particular unit, and normalize the sums to the spot surface area, thereby representing the color intensity in relative units (RU). Alternatively, a portable detector, Reflekom, equipped with Videotest software (Synteco-Complex, Moscow, Russia) was used for quantitative detection of antigen.

Based on the color intensities (*Y*) for different concentrations of the analyte (*x*), a calibration curve was constructed using the four-parameter sigmoid function [29]:

(1) $$\begin{array}{c} Y=\left(\;\frac{\left(A-D\right)}{\left(1+{\left(x/C\right)}^{B}\right)}\;\right)+D, \end{array}$$

where *A* is the asymptotic maximum (the color intensity in the absence of the analyte), *B* is the slope of the curve in semilogarithmic coordinates in the inflection point, *C* is the concentration of the analyte at the inflection point, and *D* is the asymptotic minimum (the intensity of the background coloration).

The quantitative limit of detection was calculated as the TC content corresponding to a binding inhibition of 10%. The working range was calculated as the TC content corresponding to a binding inhibition of 20% (lower limit of working range) and 80% (upper limit of working range) [30].

The visual detection limit was 1,000 RU.

### 2.12. Addition-Detection Experiments

The test system was characterized in addition-detection experiments using spiked milk samples. The recovery (*R*, %) in the addition-detection experiments was calculated as follows:

(2) $$\begin{array}{c} R,\%=\left(\;\frac{x_{\exp}}{x_{\text{theor}}}\;\right)\cdot 100\%, \end{array}$$

where *x*_theor_ is the added TC concentration and *x*_exp⁡_ is the TC concentration obtained with the calibration curve.

## 3. Results and Discussion

### 3.1. Preparation and Specification of Immunoreagents

Characteristics of immunoreagents included in the test systems included determination of the composition of hapten-protein synthesized conjugate and assessment of the interaction of the conjugate and polyclonal antibodies.

**Figure 2:**
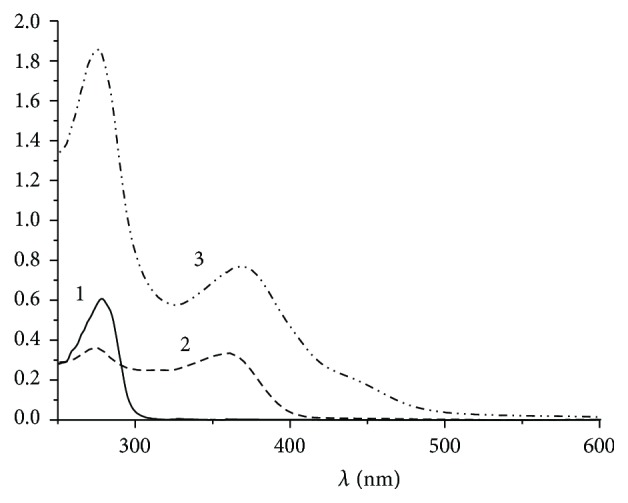
Absorbance spectra of BSA (1), TC (2), and the TC-BSA conjugate (3).

The TC-BSA conjugate was characterized using a spectrophotometer (Figure 2). According to the comparative data of the spectra of primary components and the conjugate, the molar ratio of TC : BSA in the conjugate was 10 : 1.

**Figure 3:**
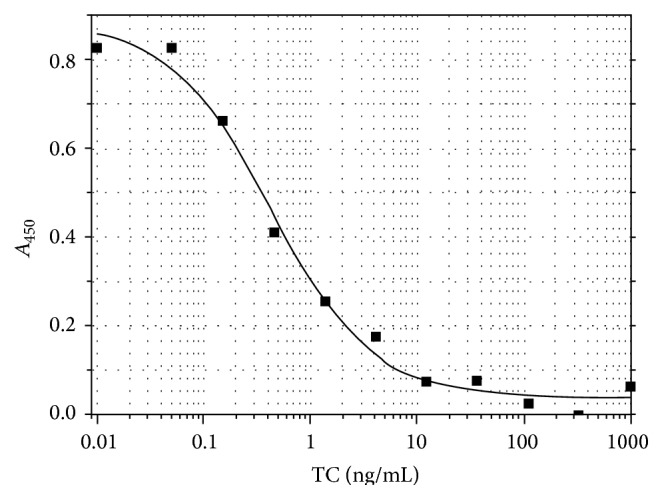
TC-competitive EIA: dependence of the recorded absorbency on the analyte concentration.

The interaction of the conjugate and polyclonal antibodies to tetracycline was characterized using the microplate enzyme immunoassay method. The concentration of antibodies absorbency of 1.0 in the microplate enzyme immunoassay to be obtained (during interaction with the immobilized TC-BSA conjugate) was 0.15 *μ*g/mL. The effectiveness of TC identification was characterized using the competitive microplate enzyme immunoassay method, the conditions of which (reactant concentration and duration of incubation) were optimized to ensure a minimum detection limit. The EIA calibration curve obtained from the selected optimal conditions (see “Section 2”) is shown in Figure 3.

As compared to control levels of tetracycline of 10–100 ng/mL, the tested polyclonal antibodies allowed its determination by means of the ELISA method at concentrations 1-2 times lower. This indicated their viability for the development of IC test systems.

### 3.2. Preparation and Characterization of Gold Conjugates for Immunochromatography

#### 3.2.1. Specification of Sizes of GNPs

Formulation of GNPs obtained by the reduction of HAuCl_4_ to Au^0^ [31] was characterized using transmission electron microscopy (TEM) and DLS methods. Two methods were used because of their different capabilities. TEM is employed to determine the size of electron-dense structures, that is, the “metal core” of the particle excluding hydrate, citrate, and other membranes. Meanwhile, the DLS method is used to characterize particles in a water environment, considering reactants immobilized on the surface and the hydration shell to approximate the obtained quantities to the properties of the reactants in a real assay environment.

**Figure 4:**
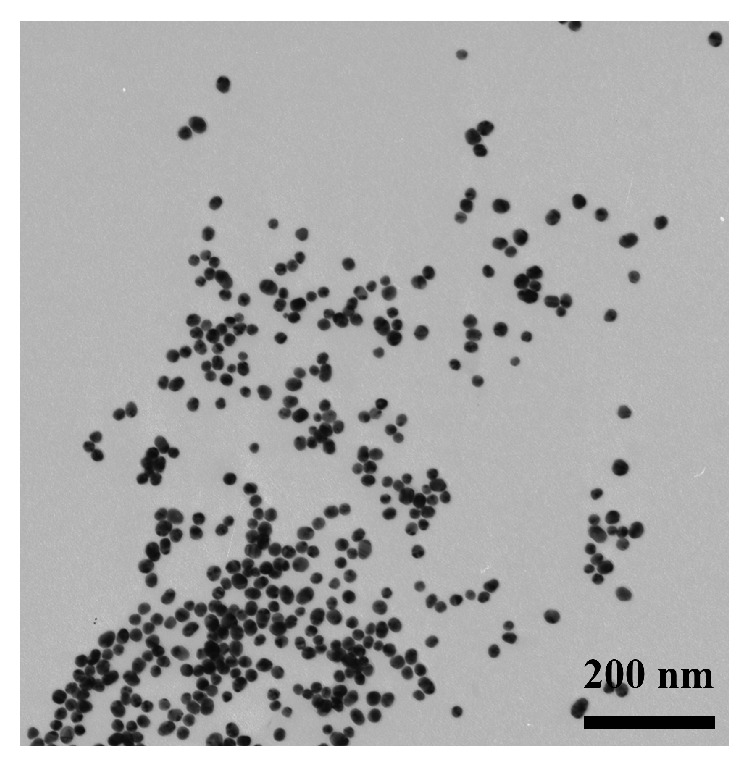
Electron microphotograph of GNPs.

The TEM showed (Figure 4) that the average diameter of the GNPs was 20.2 ± 0.8 nm. Their shape was nearly spherical, with an elongation coefficient of 1.26 ± 0.04. Based on these values, the estimated average surface area of a single GNP was equal to 13,000 ± 100 nm^2^.

**Figure 5:**
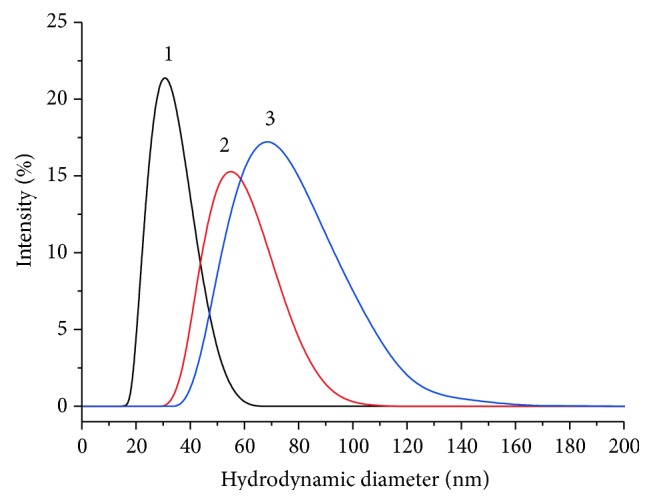
Hydrodynamic diameters for GNPs (1) and their conjugates with anti-TC antibodies, showing an antibody : GNP molar ratio of 23.5 : 1 (2) and 47 : 1 (3).

The results of DLS showed that hydrodynamic diameter of GNPs is 31.6 ± 0.3 Nm (Figure 5, 1). The differences in the values obtained by means of the TEM and DLS methods were due to the citrate shell on the surface of the GNPs. This effect and its degree of manifestation correspond to the results of our previous studies [32, 33].

The narrow monopeak particle distribution by size observed for both methods showed a homogeneous nature of the formulation of GNPs, as well as a lack of aggregates and impurities therein.

#### 3.2.2. Preparation and Characterization of Conjugates of GNPs with IgGs

**Figure 6:**
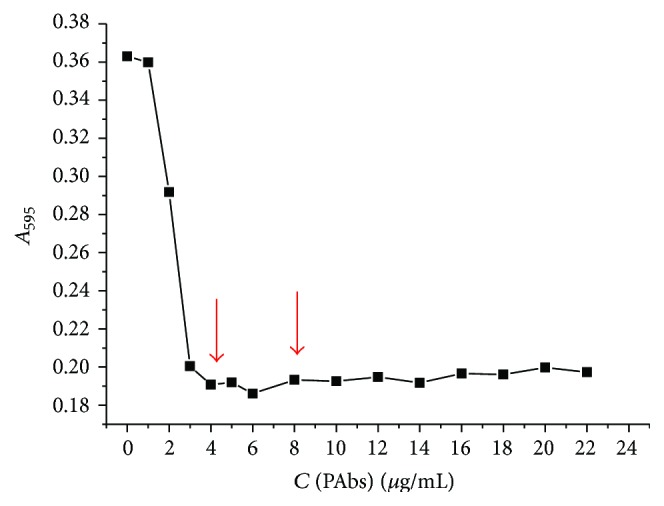
Values of GNPs optical density at 580 nm under high ionic strength obtained after GNP stabilization by the anti-TC polyclonal antibodies at different concentrations.

The conditions for the conjugation of the antibodies to GNPs were chosen based on the photometric data characterizing the aggregation of the product of this reaction at a high ionic strength. The flocculation curve was obtained through the process shown in Figure 6. For added IgG concentrations of 4 *μ*g/mL and above, there was a plateau in the recorded optical density. This demonstrates that the stabilizing nanoparticles immobilized protein molecules, preventing the aggregation of nanoparticles under conditions of high ionic strength. Based on Figure 6, the TC antibodies were taken for conjugation in an amount for the point at which OD_580_ reached the plateau and two times higher, at protein concentrations of 4.0 and 8.0 *μ*g per milliliter of the colloidal solution.

The concentration of 4 *μ*g/mL corresponded to a molar ratio of IgG : GNP during the interaction equal to 25 : 1, while the concentration of 8 *μ*g/mL corresponded to a ratio 50 : 1. Given the size of GNPs and diameter of the Fc-fragment of IgG (4 Nm), the maximum number of adsorption sites of IgG on the surface of GNP (achieved during their contact with the surface of Fc-fragment of the molecule) was 100. Thus, the amounts of IgG selected for conjugates were 26 and 52% of the maximum possible number covering the entire surface of GNP.

The accounting estimate of the immobilization process was compared with experimental data. To that effect, the concentration dependences of binding in ELISA were compared with the immobilized TC-BSA conjugate obtained for the antibody solution to be added when conjugated, as well as supernatants sampled after generating the conjugates.  Table 1 shows the experimental data and the data from calculations performed using the experimental results. As can be observed, binding of IgGs took place almost quantitatively for both formulations. Thus, at least twice the amount of IgGs (47) may be adsorbed on the GNP surface than what occurs during stabilization of the nanoparticles, in accordance with flocculation curve (24).

Dimensioning specifications for the resulting conjugates and the initial GNPs were determined by means of the TEM and DLS methods.

TEM showed an almost spherical shape of GNPs and a high degree of homogeneity. The average diameter of the conjugate with a molar ratio of IgG : GNP 24 : 1 was 21.7 ± 0.6 nm, and the elongation coefficient was 1.25 ± 0.04. The average diameter of the conjugate with a molar ratio of IgG : GNP of 47 : 1 was 22.3 ± 0.9 nm, and the elongation coefficient was 1.22 ± 0.06.

The hydrodynamic diameter of the conjugate nanoparticles varied depending upon their composition, equaling 55.4 ± 2.1 Nm for the conjugate with a molar ratio of IgG : GNP of 24 : 1 and 76.9 ± 2.8 nm for the conjugate with a molar ratio of 47 : 1 (Figure 5, curves 2 and 3).

Significant differences in size characteristics obtained by means of the TEM and DLS methods reflect the recording of membranes of immobilized molecules and bound water using the DLS method (see above). It was shown that the conjugation was not accompanied by aggregation of the nanoparticles. These findings are consistent with the literature data on the immobilization of IgG on the surface of GNPs [34–36].

**Figure 7:**
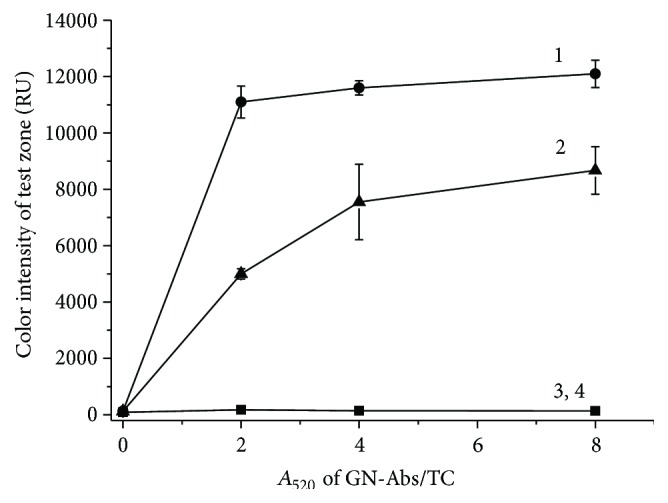
Binding of IgG-GNP conjugates (1 and 3—conjugate of composition 47 : 1; 2 and 4—conjugate of composition 23.5 : 1) with the ТС-BSA conjugate (curves 1 and 2) and standard BSA preparation (curves 3 and 4) on the immunochromatographic membrane.

Preservation of functional activity by IgG-GNP conjugates was tested by their binding to the TC-BSA conjugate on the immunochromatographic membrane (Figure 7). The TC-BSA conjugate was replaced with native BSA conjugate to confirm the specific nature of interaction. As follows from Figure 7, the conjugates showed comparable (at equal values of absorbency) degrees of binding of a specific nature, which allows their use in the development of IC test systems.

### 3.3. Development of an Immunochromatographic Assay for Tetracycline

The immunochromatographic assay protocol was optimized to achieve a minimum TC detection limit, including selection of detergent concentration, concentrations of IgG-GNPs and TC-BSA immobilized conjugates, and preparation of milk samples. The assay optimization using the IgG-GNPs conjugate of 47 : 1 is described below. The IC test system implemented based on an IgG-GNP conjugate of 24 : 1 showed the worst analytical characteristics; therefore, it was excluded from further study.

**Figure 8:**
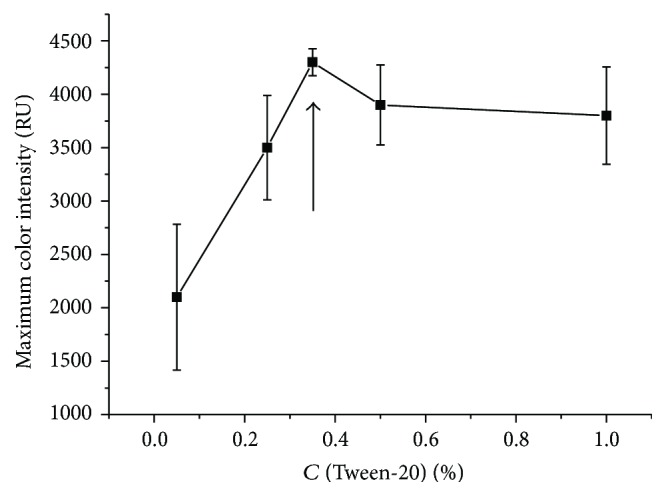
Dependence of the intensity of coloration of the test zone of IC test systems on Tween-20 detergent concentration in the absence of TC.

Use of detergent in immunochromatography (added to the IgG-GNP conjugate when applied) promotes uniform rapid movement of liquid along the membranes of the test strip, the most complete elution of IgG-GNP conjugate, and an increase in the intensity of coloration of the test zone [36]. The results obtained with various concentrations of Tween-20 detergent are shown in Figure 8. The concentration range under analysis was 5–100 times greater than the critical concentration for micelle formation (CCM) of Tween-20, which was equal to 0.01% [37]. As follows from the results, a Tween-20 concentration of 0.35% provided a uniform movement of the liquid front, clear boundaries of the binding zone, and maximum intensity of coloration. This concentration, which was chosen as optimal, is 35 times greater than the CCM. The detergent concentrations recommended in the literature and used in the immunoassay were at least 5 times greater than the CCM [38, 39].

The IgG-GNP conjugate concentration was varied in the range corresponding to the variation of OD_580_ from 2 to 8 absorbency units. As follows from the data obtained (Table 2), the maximum intensity of coloration was constant in this range (i.e., coloration in the absence of analyte in the sample) within the tolerance. For the assay option, OD_580_ = 4 was optimal. This dilution provided a minimum detection limit and minimal background coloration of the test zone.

Maximum intensity of coloration reaching 4,300 ± 35 units at 2 mg/mL was increased with an increase in the TC-BSA conjugate concentration in the test zone. Substantial background coloration was typical for higher concentrations. The optimal TC-BSA conjugate concentration was therefore selected as 2 mg/mL.

**Figure 9:**
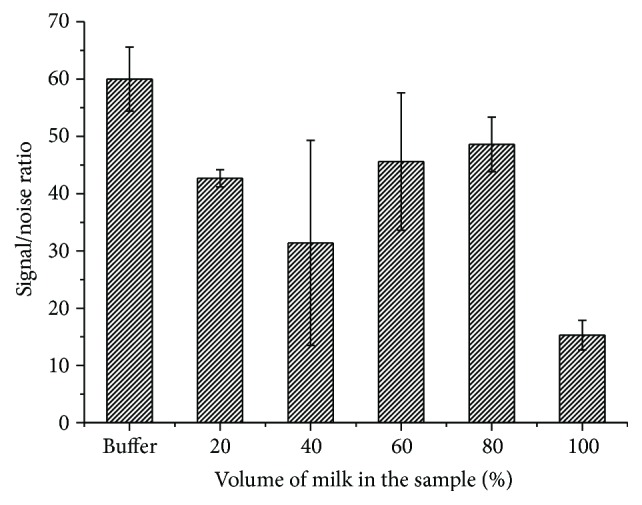
The dependence of the intensity of coloration of the test zone of IC test systems in the absence of TC on the degree of dilution of milk samples using buffer (PBS).

As sample preparation should be maximally simple, rapid, and non-time-consuming in the IC assay, milk samples were diluted with buffer (PBS) for this purpose. The results obtained for different variants of dilution are shown in Figure 9. The optimal volume ratio of the sample and the buffer was 4 : 1 (80% content of milk in the test sample). In this case, the sample matrix had minimal effect on the intensity ratio of specific and background coloration, reaching 50 : 1, and the TC detection limit remained unchanged. The use of a large sample dilution would reduce the sensitivity of the test system.

Several parameters of the IC test system, including membranes used, were selected based on our previous data on the development of test systems for other antigens [24, 28, 40].

The test strips used in further study were produced in accordance with the optimal immunochromatographic parameters.

### 3.4. Testing of the Developed Immunochromatographic Test System

**Figure 10:**
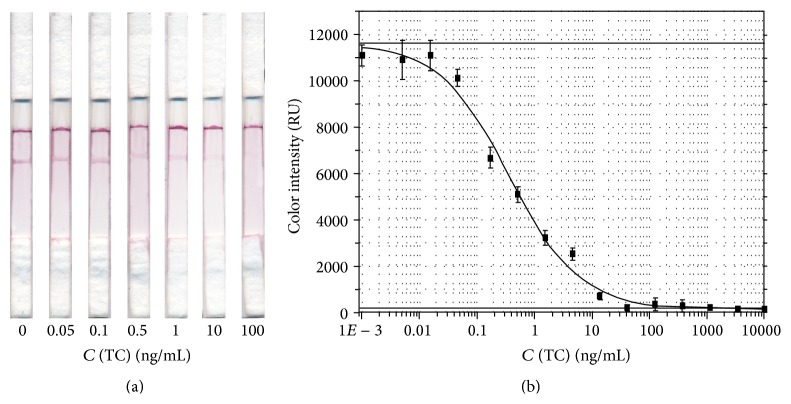
Test strips (a) and calibration curve, *n* = 3 (b) for immunochromatographic determination of the TC in milk samples.

A calibration curve was obtained to identify TC in milk.  Figure 10 shows the results of the testing of samples containing different concentrations of ТС in milk. The test system developed is characterized by low instrumental detection threshold (0.02 ng/mL) and a wide operating range (0.06–10 ng/mL). The operating range covering more than two orders of concentrations is considerably higher than the range provided in the test systems based on monoclonal antibodies (one order) [41]. This effect was achieved through the use of polyclonal antibodies characterized by a considerable variability of individual clones by affinity, thereby providing a competitive interaction in a wide range of concentrations. The precision (standard deviation) of the concentrations measurements was no more than 10%. The disappearance of the color in the test zone corresponded to the Russian MRL of TC (10 ng mL^−1^). The dependence of color intensity from TC concentration is described by the following equation:

(3) $$\begin{array}{c} Y=\left(\;\frac{\left(11640-158.8\right)}{\left(1+{\left(x/0.36\right)}^{0.7}\right)}\;\right)+158.8. \end{array}$$

Adequate goodness of fit (*χ* ^2^) for this equation is 5.3 and *R* ^2^ is 0.996.

The test system developed was validated to identify TC in milk samples. This is characterized in addition-detection experiments. The data shown in Table 3 are indicative of the efficiency of the test system as a means of quantitative assay; the degree of TC extraction was 102 ± 8%.

## 4. Conclusions

The developed IC test system is characterized by a low detection threshold of TC in milk of 0.02 ng/mL for instrumental recording, as well as a wide (more than two orders) operating range. The quantitative assay method proposed is 10–100 times more sensitive than IC assay [21] and ELISA [18, 42]. The visual detection threshold of the test system is 10 ng/mL, which is 2–5 times greater than that of commercial analogues (SNAP, Charm). The assay time is 10 min, including non-time-consuming sample preparation, which is limited to dilution of the sample under analysis using a buffer. The effectiveness of the test system for monitoring tetracycline in milk was confirmed. The characteristics established allow the developed test system to be considered as a promising tool for monitoring the safety of foods.

Detection threshold reduction was achieved using two methods, as follows: (a) transition from a qualitative assessment of results to a quantitative assessment based on video digital recording and (b) the use of a polyclonal formulation containing a small proportion of antibodies specific to TC and nonspecific antibodies stabilizing GNPs. These approaches are universal and can potentially be used in the development of IC test systems to identify a wide range of compounds.

## Figures and Tables

**Table 1:** Antibody immobilization characteristics on the GNP surface.

Concentration of antibodies when conjugated, *µ*g/mL	4.0	8.0
Molar ratio of IgG : GNP when conjugated	25 : 1	50 : 1
Residual concentration of antibodies determined in the supernatant, *µ*g/mL	0.22	0.47
Antibody immobilization degree, %	94.5	94.1
Actual molar ratio of IgG : GNP in the conjugate	24 : 1	47 : 1

**Table 2:** The dependence of TC immunochromatographic assay on IgG-GNP conjugate concentration.

OD_520_ of the conjugate	Limit of TC detection, ng/mL	Maximal coloration, RU	Background coloration, RU
2	0.16 ± 0.06	11,100 ± 568	270 ± 58
4	0.1 ± 0.02	11,600 ± 251	170 ± 25
8	1.8 ± 0.18	12,000 ± 485	5,000 ± 186

**Table 3:** Description of the test system developed for TC for testing milk samples in “addition-detection” experiments.

TC added concentration, ng/mL	TC concentration established by means of the test system, ng/mL	Recovery^*∗*^, %
0.03	0.027 ± 0.001	90 ± 3
0.3	0.29 ± 0.01	102 ± 8
0.5	0.56 ± 0.02	112 ± 4
1.5	1.48 ± 0.04	96 ± 3
2	2.2 ± 0.1	110 ± 7

^*∗*^ *n* = 3.
